# Autophagy-related prognostic signature for survival prediction of triple negative breast cancer

**DOI:** 10.7717/peerj.12878

**Published:** 2022-02-09

**Authors:** Qiong Yang, Kewang Sun, Wenjie Xia, Ying Li, Miaochun Zhong, Kefeng Lei

**Affiliations:** 1 Department of General Surgery, Cancer Center, Division of Breast Surgery, Zhejiang Provincial People’s Hospital, People’s Hospital of Hangzhou Medical College, Hangzhou, China; 2 Department of General Surgery, The 7th Affiliated Hospital of Sun Yat-sen University, Shenzhen, China

**Keywords:** Autophagy gene, Prognosis, Predict model, Triple negative breast cancer, TCGA

## Abstract

**Background:**

Triple-negative breast cancer (TNBC) is a highly aggressive type of cancer with few available treatment methods. The aim of the current study was to provide a prognostic autophagy-related gene (ARG) model to predict the outcomes for TNBC patients using bioinformatic analysis.

**Methods:**

mRNA expression data and its clinical information for TNBC samples obtained from The Cancer Genome Atlas (TCGA) and Metabric databases were extracted for bioinformatic analysis. Differentially expressed autophagy genes were identified using the Wilcoxon rank sum test in R software. ARGs were downloaded from the Human Autophagy Database. The Kaplan–Meier plotter was employed to determine the prognostic significance of the ARGs. The sample splitting method and Cox regression analysis were employed to establish the risk model and to demonstrate the association between the ARGs and the survival duration. The corresponding ARG-transcription factor interaction network was visualized using the Cytoscape software.

**Results:**

A signature-based risk score model was established for eight genes (*ITGA3*, *HSPA8*, *CTSD*, *ATG12*, *CLN3*, *ATG7*, *MAP1LC3C*, and *WIPI1*) using the TCGA data and the model was validated with the GSE38959 and Metabric datasets, respectively. Patients with high risk scores had worse survival outcomes than those with low risk scores. Of note, amplification of *ATG12* and reduction of *WIPI* were confirmed to be significantly correlated with the clinical stage of TNBC.

**Conclusion:**

An eight-gene autophagic signature model was developed in this study to predict the survival risk for TNBC. The genes identified in the study may favor the design of target agents for autophagy control in advanced TNBC.

## Introduction

Breast cancer (BC) is the most lethal malignancy among women (Fitzmaurice et al., 2019). Approximately 15% of BC cases are triple-negative breast cancer (TNBC). TNBC is a highly heterogeneous and highly aggressive BC subtype characterized by the lack of expression of hormone receptors (HR) and human epidermal growth factor receptor 2 (HER2) (Lehmann et al., 2011; Sorlie et al., 2001). Standard chemotherapy remains the primary treatment option for TNBC patients (Anders & Carey, 2008; Prat et al., 2015). Though new target agents such as poly (ADP-ribose) polymerase inhibitors, phosphoinositide 3-kinase inhibitors, and immune checkpoint inhibitors have shown promising efficacy in prolonging the overall survival (OS) times, most of these agents benefit only small populations of patients harboring specific gene changes (Lehmann et al., 2014; Narod, Booth & Foulkes, 2017; Yonemori et al., 2019). Most patients who experience disease progression therefore struggle with the lack of effective drugs, and the median OS is estimated to be only 18 months.

Autophagy is an intracellular evolutionarily conserved, self-degradation process, in which cytoplasmic macromolecules, damaged proteins, organelles, or pathogens are delivered to lysosomes and digested by lysosomal hydrolases (Klionsky, 2007; Nakatogawa et al., 2009; Yorimitsu & Klionsky, 2005). Due to this degradation and renovation process, autophagy is essential for the maintenance of homeostasis in the physiological condition and plays vital roles in the development of many diseases, such as cardiovascular disorders, neurodegeneration, autoimmune diseases, and cancer (Ren, Sowers & Zhang, 2018; Ye et al., 2019; Yin et al., 2018). Since autophagy provides cell fuel such as essential metabolites or amino acids to tumor cells under stress, the process may sustain and promote tumor growth. Gene mutations abrogate the autophagy capability of eukaryocytes, making it easier for cancer cells to develop more autophagy blockage than normal cells. It is now recognized that autophagy can both impede and promote carcinogenesis (Bortnik & Gorski, 2017; Goldsmith, Levine & Debnath, 2014; Singh et al., 2018).

TNBC cells exhibit dysregulation in many autophagic signal pathways. Combined use of autophagy inhibitors and other chemotherapy drugs results in a synergistic effect and may reverse drug resistance (Lefort et al., 2014; Park et al., 2016; Rontogianni et al., 2020). Herein, to understand the autophagy genes critical for TNBC development, data currently available in public databases, specifically the Cancer Genome Atlas (TCGA), and the Human Autophagy Database (HADb), were compiled to identify differentially expressed genes (DEGs) related to autophagy in TNBC. Using a sample splitting method and Cox regression analysis, a gene risk score model was established in this study to assess the association between autophagy-related genes (ARGs) and the prognosis of TNBC. The model is expected to be applicable for prognostic evaluation in clinical settings, to provide potential targets capable of modulating cancer-related autophagic pathways, and to improve the survival of TNBC patients.

## Materials and methods

### Gene expression profile data from GEO and TCGA

TNBC microarray data (GSE38959) (Komatsu et al., 2013) were downloaded from the GEO database. The dataset met the following criteria: (1) it contained data on patients with TNBC; (2) it contained case-control groups; and (3) it contained at least 30 samples.

mRNA expression data along with the clinical information (including survival state, survival time, age, pathological stage, TNM stage) from a cohort of 111 patients with TNBC and 10 matched non-cancerous samples were retrieved from TCGA. mRNA expression was quantified based on the Genome Research Project of the Encyclopedia of DNA Elements (GENCODE, GRCh38 catalog; http://www.gencodegenes.org/). The raw data were normalized and analyzed using the edgeR package (Robinson, McCarthy & Smyth, 2010).

### Distinct autophagy-related gene expression profiles

ARGs were downloaded from the HADb. The genes identified from bioinformatics analysis of the TCGA dataset were evaluated for intersections with genes in the HADb database. Overlapping genes were deemed autophagy genes that were expressed in TNBC and were retained for further survival and functional analyses. Additionally, the identification of differentially expressed autophagy genes was performed using the Edge Package in R software (3.6.0). Genes with —log2FC—>1 and adjusted *P*-values <0.05 were considered to be statistically significant. Heatmaps were generated by hierarchical clustering analysis and used to identify differences in gene expression. The ggpubr package in R software was used to generate a box plot.

### Construction of risk score model for survival

To determine the genes that could be used for survival prediction, the correlations between the survival information for TNBC and the expression of autophagy-related genes were analyzed using the univariate Cox proportional regression model (O’Quigley & Moreau, 1986) with *P* < 0.05 set as the significance threshold. To prevent over-fitting, LASSO regression analysis was conducted to screen out autophagy genes significantly related to the prognosis. Based on the correlation coefficients obtained with LASSO regression (Fontanarosa & Dai, 2011), a risk prediction model was constructed: risk score = ΣCoef_mRNAs_ ×Exp_mRNAs_, where Coef_mRNAs_ represents the regression coefficient, and Exp_mRNA_ indicates the expression level of the corresponding mRNA. The risk score for each sample was calculated using the above formula. Using the median of the risk scores as the cut-off value, the samples were divided into high-risk and low-risk groups. Using the timeROC package in R software (https://cran.r-project.org/web/packages/timeROC/index.html) (Heagerty, Lumley & Pepe, 2000), the feasibility and reliability of the prognosis model at 1, 3, and 5 years were evaluated.

### Survival significance of the model

To assess whether the risk score system can be used as an independent prognostic factor for TNBC, the risk score obtained from the model was associated with the clinical characteristics of TNBC patients (age and pTNM stage) using univariate and multivariate Cox regression analyses. Forest maps were drawn to represent the relationship and the disease prognosis was analyzed using the “Survival” package in R software (https://cran.r-project.org/web/packages/survival) (O’Quigley & Moreau, 1986) with a significance threshold for *P* values < 0.05.

### Target regulatory network analysis

Cancer transcription factor (TF) data were downloaded from the Cistrome database. The corresponding expression levels for TFs in TNBC were extracted from TCGA. The correlations of TFs with the eight prognosis-related genes were analyzed and the correlation coefficient for TF screening was set as >0.3 with *P* < 0.01 (Liu et al., 2011; Shannon et al., 2003). One hundred and fifty seven interaction relationships were obtained and visualized using the Cytoscape website.

### External validation

The validation dataset (GSE38959) was downloaded from the GEO database. Receiver operating characteristic (ROC) (Le et al., 2019a; Le, Yapp & Yeh, 2019b) analysis was used to evaluate whether the prognostic genes had good pattern recognition between the tumor and normal tissue. We also downloaded another dataset (Metabric) from the cBioPortal database (https://www.cbioportal.org/) and the Kaplan–Meier curve was adopted to identify the survival difference between risk groups.

### Statistical analysis

Univariate and multivariate Cox proportional hazards regression analyses were conducted using the “Survival” package in R software. The hazard ratio (HR) and 95% confidence interval (CI) were calculated to identify protective (HR <1) and risk-related genes (HR >1). A survival curve was plotted with the Kaplan–Meier (KM) method to estimate the differences in the survival duration between the high- and low-risk patients. All the statistical analyses were conducted with R (version 3.6.0).

## Results

### Preliminary screening of differentially expressed ARGs from TCGA

**Figure 1:**
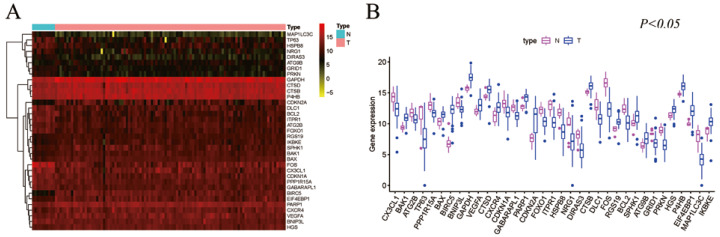
Differentially expressed autophagy genes in TNBC from the TCGA database analysis. (A) The expression pattern of 35 genes in TNBC. Red indicates higher expression and yellow indicates lower expression. (B) The boxplot of 35 genes identified from the TCGA dataset.

Gene expression profiles for 111 TNBC samples, composed of 101 primary TNBC samples and 10 normal breast tissue samples with clinical files, were obtained from TCGA. The HADb was also searched and 232 genes implicated in the autophagy process were extracted. A total of 35 differentially expressed ARGs (DE-ARGs) in TNBC were retrieved from the TCGA database (log2FC >1 and *P* < 0.05). The validated top five up-regulated genes were BIRC5, CDKN2A, EIF4EBP1, GAPDH, and IKBKE, while the top five down-regulated hub genes were FOS, MAP1LC3C, TP63, DLC1, and HSPB8 (Fig. 1).

### Survival analysis of the candidate autophagy genes

**Figure 2:**
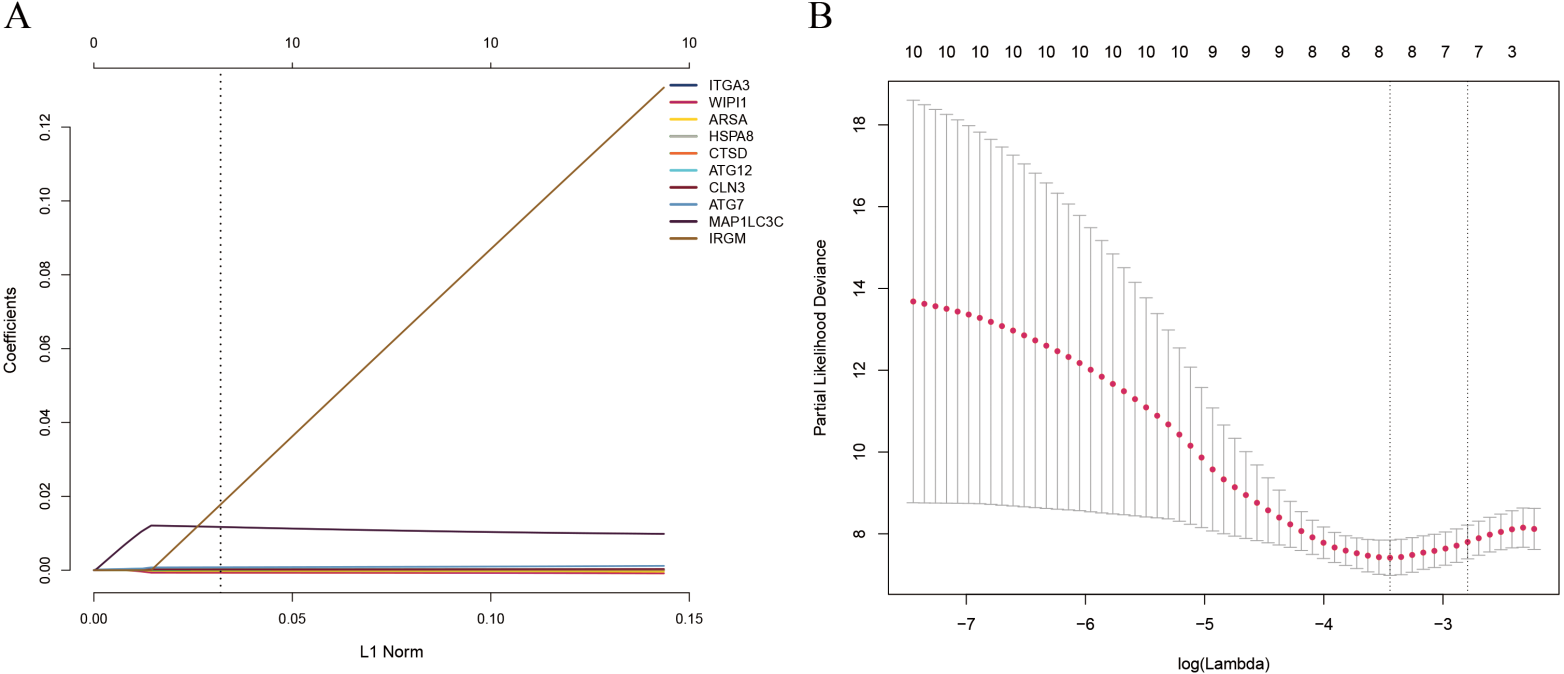
(A) LASSO coefficient profiles of the 10 survival-associated genes. (B) The tuning parameter (*λ*) in the LASSO model selected through a cross-validation procedure was plotted as a function of log(*λ*). The y-axis represents the partial likelihood dev.

**Figure 3:**
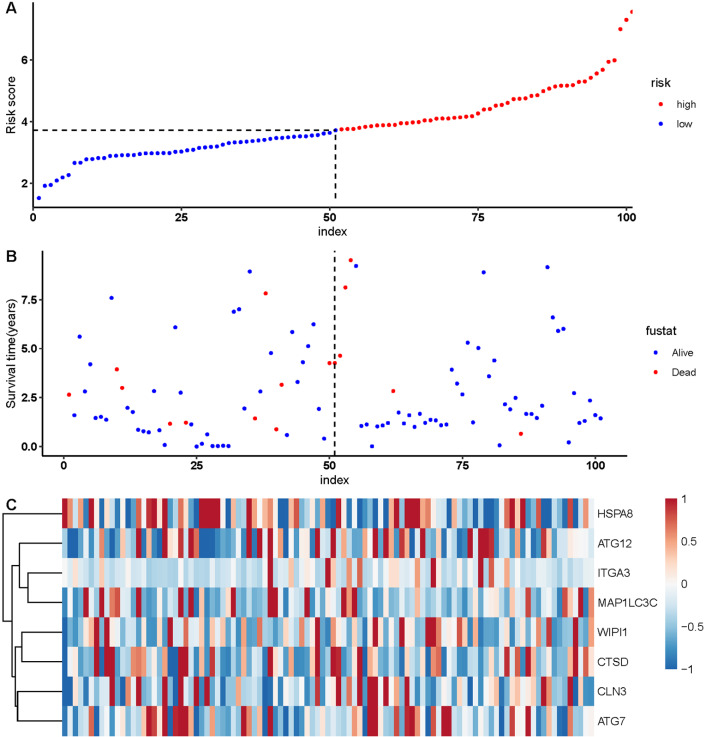
Autophagy-related gene signature risk score distribution and heat-map of the eight gene expression profiles. (A) Patients were classified into high- and low-risk groups using the median of the risk scores as the cut-off value. (B) Death events occurred more frequently in the high-risk group. (C) Heat-map of the ARG expression profiles: Rows represent genes and columns represent patients. Red indicates higher expression and blue indicates lower expression.

**Figure 4:**
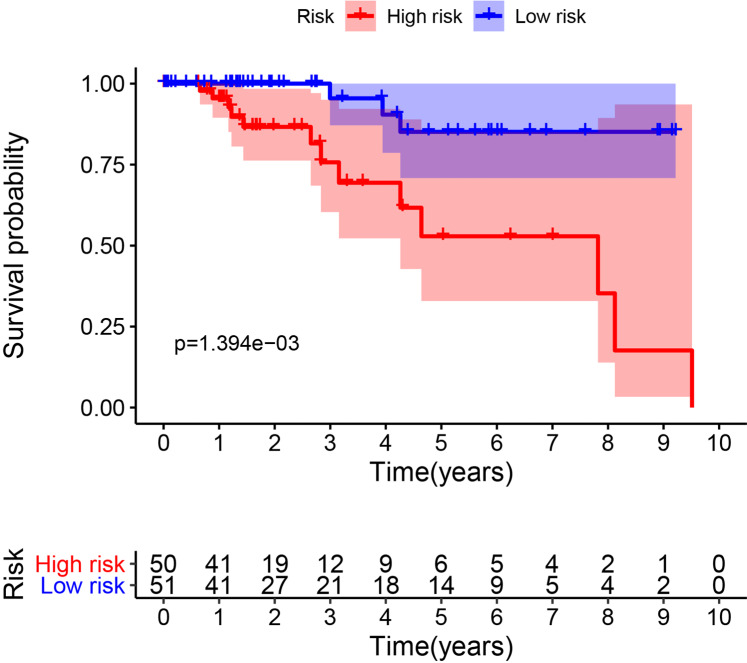
Survival analysis for the eight gene signatures between the high-risk and low-risk groups.

Since the number of DE-ARGs was small, all of the autophagy genes were included in the survival analysis. The expression levels of autophagy genes and their clinical data were extracted from the corresponding database for survival analysis. Univariate Cox regression analysis revealed 10 ARGs with significant prognostic value (*P* < 0.05) (Table 1). Among these, eight optimal gene sets were selected using LASSO regression analysis (Figs. 2A and 2B) to construct a risk score system for TNBC. These genes were integrin alpha-3 (*ITGA3*), heat shock protein family A member 8 (*HSPA8*), cathepsin D (*CTSD*), autophagy-related 12 (*ATG12*), neuronal ceroid lipofuscinosis (*CLN3*), *ATG7*, microtubule associated protein 1 light chain 3 gamma (*MAP1LC3C*), and WD repeat domain phosphoinositide-interacting protein 1 (*WIPI1*). Based on the Cox coefficients and mRNA expression levels for individual genes, the following formula was used to calculate the risk scores for each sample: risk score = 2.50E-05*ITGA3 + −0.000109582*WIPI1 + 2.40E−05*HSPA8 + 2.11E−06*CTSD + 0.000372025*ATG12 + 0.000170205*CLN3 + 0.000365579*ATG7 + 0.008241647*MAP1LC3C. With the median of the risk scores set as the cut-off value, the cut off value was 41.11, the patients were classified into high- and low-risk groups (Fig. 3A). As shown in Fig. 3B, death events occurred more frequently in the high-risk group than in the low-risk group. The expression status of the genes in patients is presented individually as dots with specific colors in Fig. 3C. Additionally, the KM curves showed that patients in the low-risk group survived longer than those in the high-risk group (Fig. 4; *P* < 0.05).

**Table 1:** Autophagy genes independently related to TNBC prognosis.

ID	HR	Hazard ratio	95% CI	*P* value
ITGA3	1.000044	1.000012	1.000076	0.007217
WIPI1	0.999282	0.998627	0.999938	0.03193
ARSA	1.000152	1.000013	1.000292	0.032175
HSPA8	1.000033	1.000012	1.000053	0.001653
CTSD	1.000016	1.000006	1.000026	0.001976
ATG12	1.000598	1.000044	1.001151	0.034299
CLN3	1.000413	1.000023	1.000803	0.038094
ATG7	1.000834	1.000319	1.001349	0.00149
MAP1LC3C	1.013562	1.00074	1.026548	0.038099
IRGM	1.117321	1.026867	1.215744	0.01001

**Figure 5:**
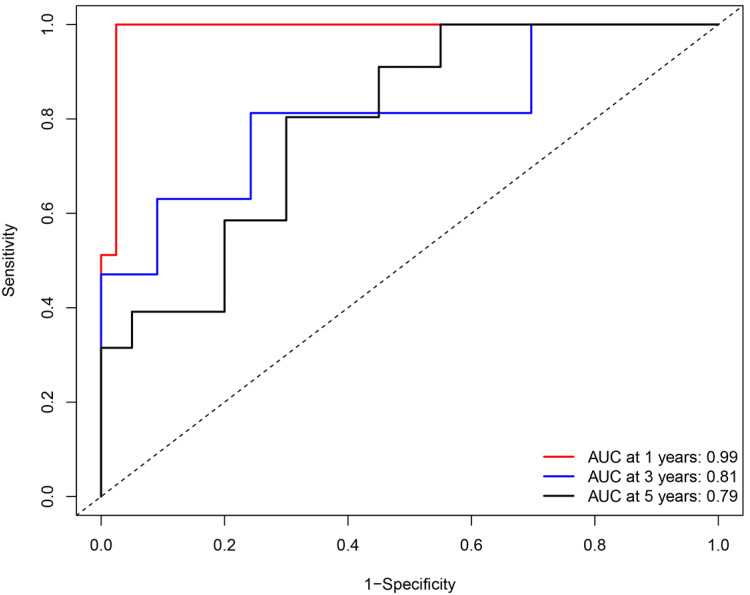
Receiver operating characteristic (ROC) analysis of the sensitivity and specificity of the survival duration predicted by the eight-gene signature-based risk score in 1, 3, and 5 years.

A ROC curve was plotted to evaluate the reliability of this prognostic model. The AUC values for 1, 3, and 5 years were calculated as 0.99, 0.81, and 0.79 respectively, which indicated the performance of this prognostic model was reliable in the clinical setting (Fig. 5).

### Prognosis significance for the risk model

**Figure 6:**
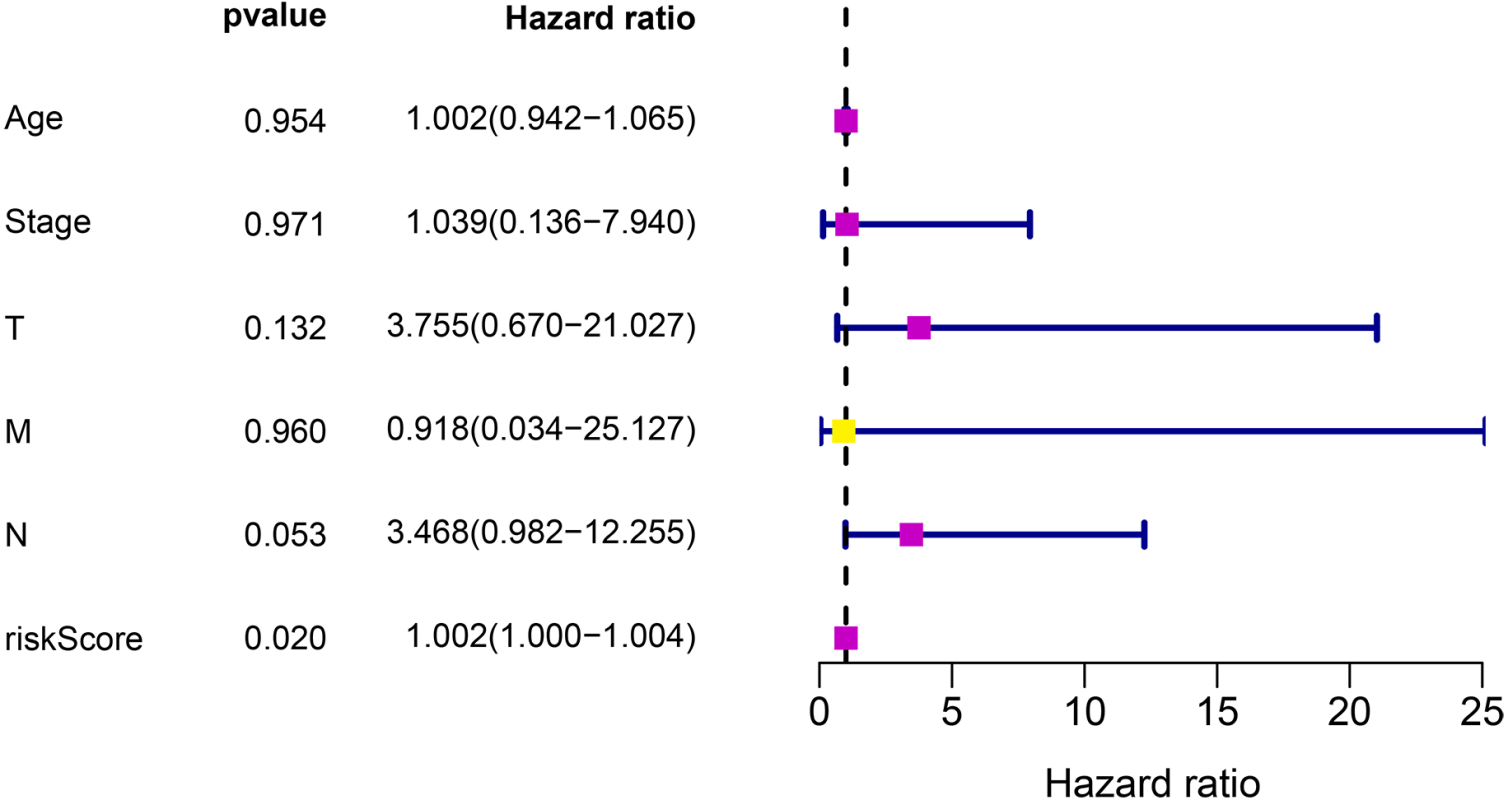
Cox regression analysis for the clinical factors and the eight-gene risk score.

To investigate whether the risk prediction system can be used as an independent prognostic factor, the relationships between the risk scores obtained from the model and the clinical characteristics of the patients were evaluated. The results confirmed that the risk score model can be used as an independent prognostic factor to predict the survival of TNBC patients (*P* < 0.05) (Fig. 6).

### Transcription factor correlation analysis

**Figure 7:**
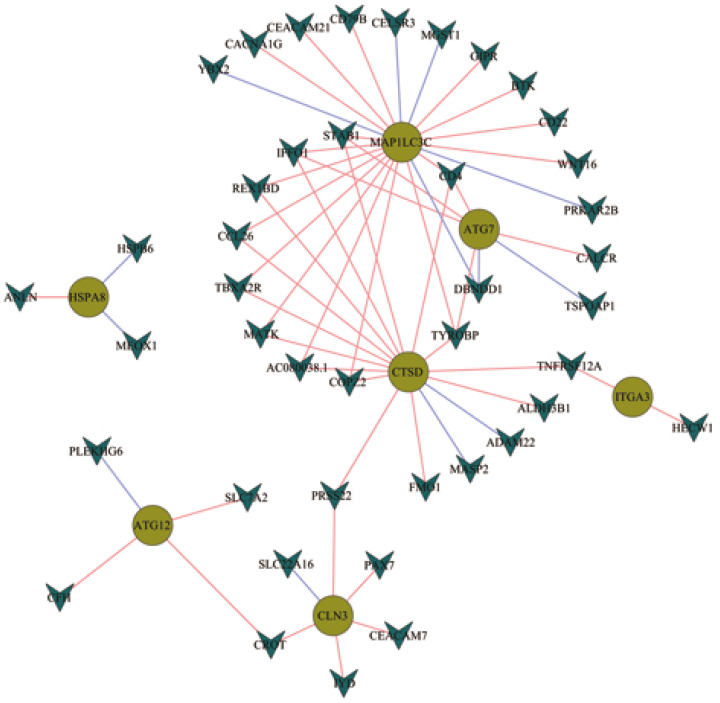
Network construction for the transcription factors and prognostic autophagy genes.

Data for 318 TFs were downloaded from the Cistrome database. Expression files for the TFs were extracted from TCGA and 61 interaction relationships for seven of the eight optimal gene sets were identified through combined analysis. The interaction networks for these genes were visualized using the Cytoscape software (Fig. 7). CTSD, ATG7, and MAP1LC3C were located at the core positions in the network, which indicated that they may share common regulatory genes and may participate in signal pathways with similar functions.

### Clinical significance of the autophagy gene sets

**Figure 8:**
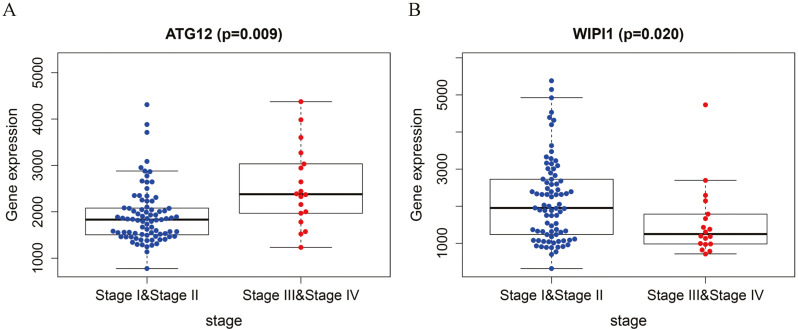
Correlation analysis for the prognostic genes and clinical traits.

Analyzing the correlations between the expression levels of the eight genes and the clinical characteristics of the patients revealed that the expression levels of *ATG12* and *WIPI1* were significantly associated with the pathological stage of TNBC. *ATG12* levels in stage I-II patients were significantly lower than those in stage III-IV patients (*P* = 0.009). The *WIPI1* levels in stage I-II patients were significantly higher than those in stage III-IV patients (*P* = 0.02). This indicated that clusters for the tumor stage could be created based on the two genes with significant differences, providing new avenues for the prognostic prediction of TNBC (Fig. 8).

### Premium prognostic model evaluation

**Figure 9:**
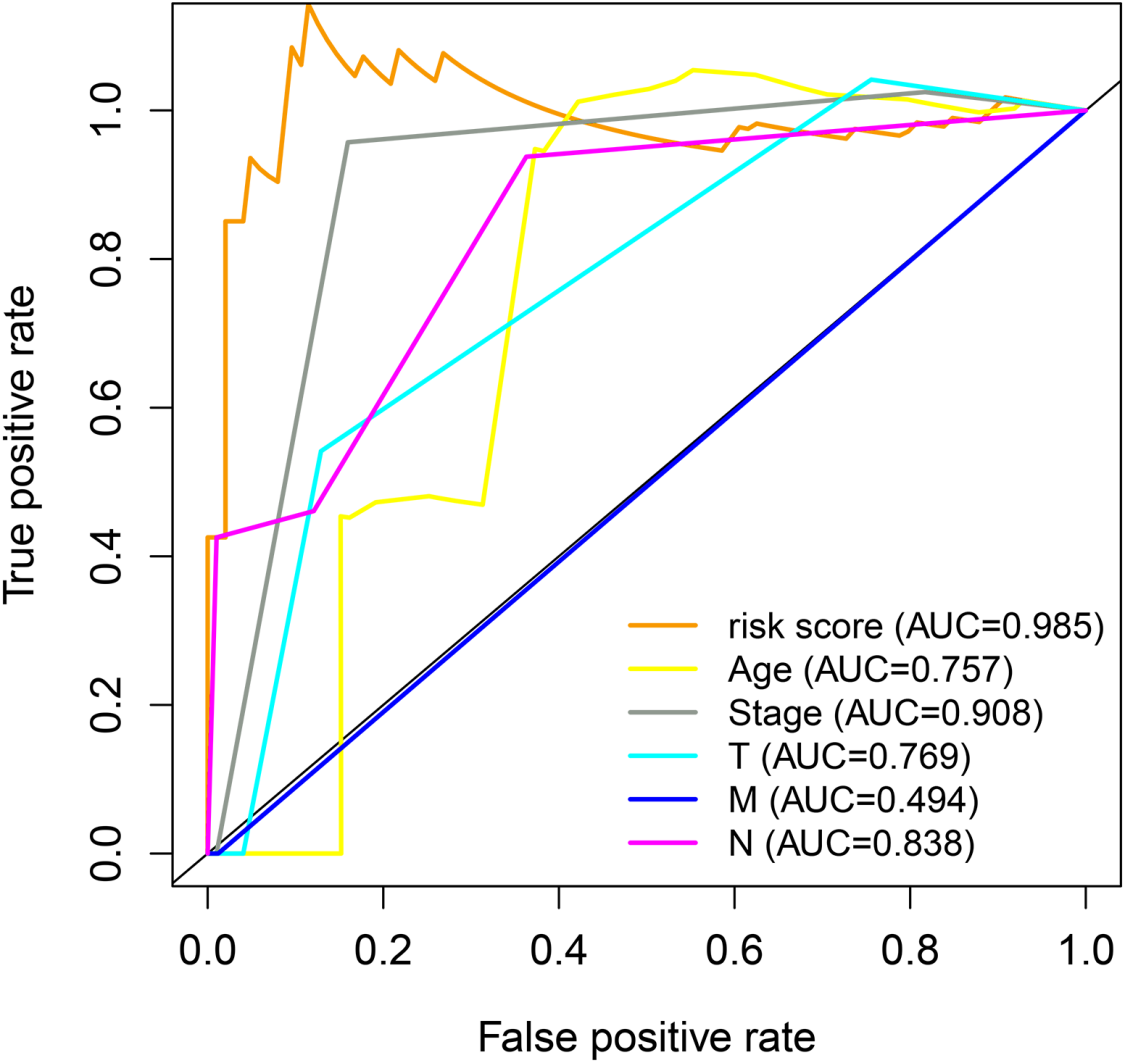
ROC analysis for the prognostic genes and clinical traits.

The association between the clinical characteristics of the samples and the risk score from the prognosis model was investigated. As shown in Fig. 8, the area under the curve (AUC) value for the risk score was higher than that for clinical parameters such as age, clinical stage, and TMN stage, indicating the gene signature model has excellent performance for survival length estimation (Fig. 9).

### External validation

**Figure 10:**
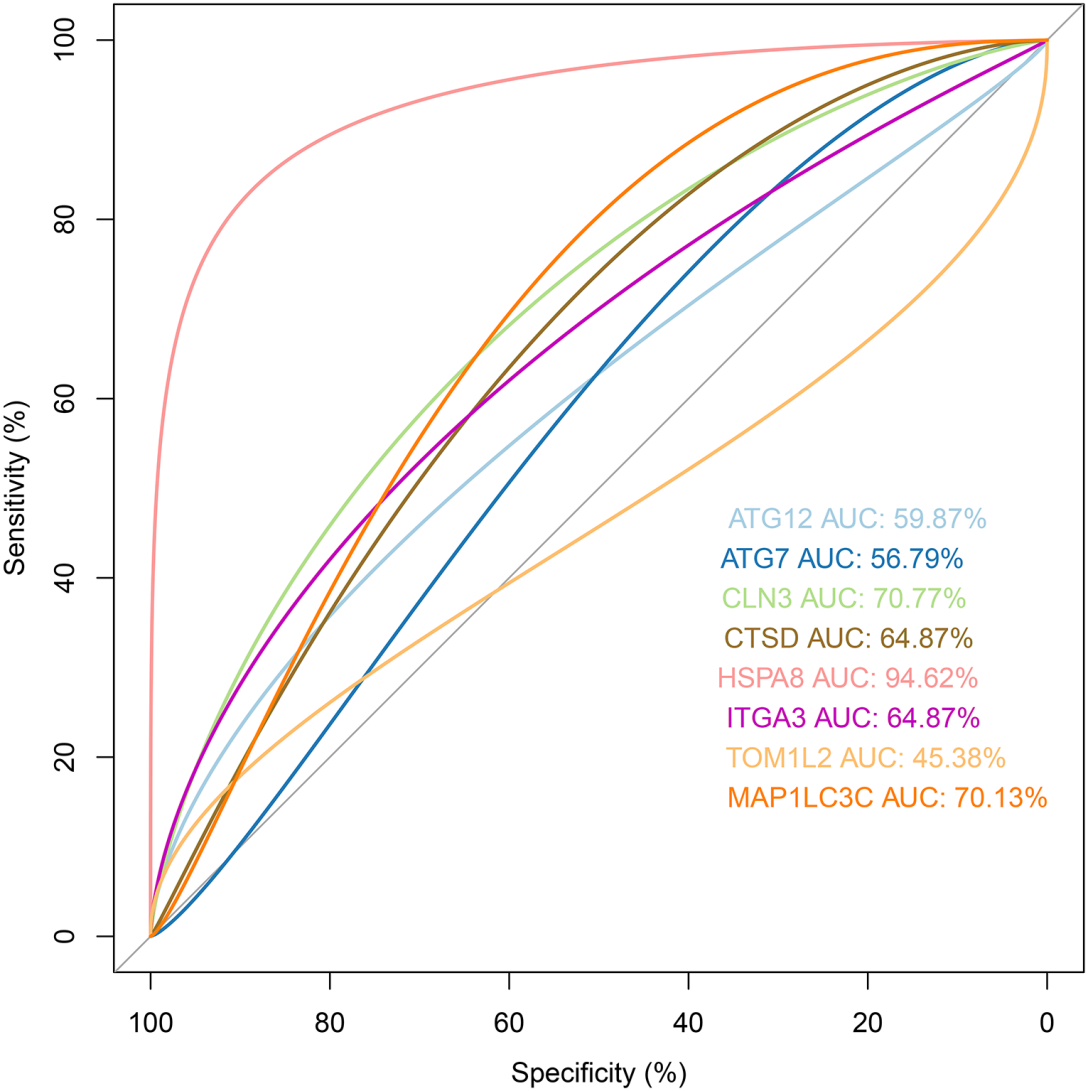
ROC analysis for the eight prognostic genes in the validation dataset.

**Figure 11:**
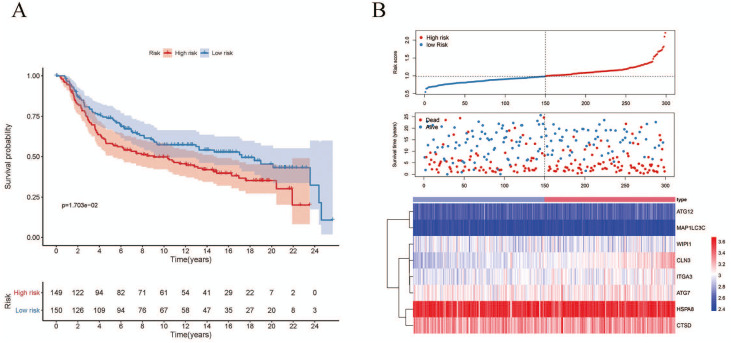
Associations between the autophagy-related risk signature and survival features in TNBC patients from analysis of the Metabric dataset. (A) Kaplan–Meier curves for OS in the high-risk and low-risk groups when stratified according to the autophagy-related signature (*P* < 0.05); (B) Based on the median risk score, the eight-gene risk signature was used to divide patients into high-risk and low-risk groups with distinct prognoses in the Metabric validation set. The red dots represent deaths, while the blue dots represent living patients. In the heat map, the transition from blue to red suggests that the expression level increased.

The validation dataset (GSE38959) downloaded from the GEO database comprised 30 TNBC samples and 13 normal breast tissue samples. The expression levels of the prognostic genes and their corresponding clinical information extracted from GSE38959 were used to confirm the reliability of the prognosis model. ROC analysis was then performed to verify the degree of pattern recognition for the genes. The prediction accuracy of the genes was proportional to the AUC value (Fig. 10). These results suggested that the eight-gene-based classifier could accurately indicate the diagnosis for TNBC patients. In addition, we used the Metabric dataset to validate the performance of the signature. As shown in Fig. 11A, the KM curve analysis suggested that a significant survival divergence existed between the high-risk and low-risk groups (*P* < 0.05). We also discovered that the high-risk group had more death cases and high risk scores, whereas patients in the low-risk group had better survival durations (Fig. 11B).

## Discussion

Autophagy, also known as type II cell death, serves as a system for intracellular control and enables cells to adapt to stress and adverse surroundings; thus, the autophagic response defends the body against diseases (He & Klionsky, 2009; Khandia et al., 2019). In some situations, autophagic dysfunction can cause malignant transformation and degeneration, particularly in conditions such as hypoxia and malnutrition. Individuals with defects or dysregulation in autophagic genes may develop various malignancies (Chen et al., 2019; Debnath, 2011; Galluzzi, Pedro & Kroemer, 2016). Autophagy in cancer-associated fibroblasts has been reported to cause TNBC cells to engage in the epithelial–mesenchymal transition process through Wnt/ *β*-catenin, resulting in enhanced cancer cell invasion and proliferation (Wang et al., 2017). Compounds administered to elicit mitochondrial dysfunction in MDA-MB-231 cells can induce the production of reactive oxygen species and suppress cell growth (D’Anneo et al., 2013). Further, deubiquitinase inhibitors impair TNBC viability and lead to the activation of autophagy, which compensates for stress in the ubiquitin-proteasome system (Vogel et al., 2015).

In this study, a viable panel of ARGs specifically expressed in TNBC was identified based on integrin bioinformatic analysis of data in the public *TCGA* and HADb databases. We noticed that the top five upregulated and top five downregulated genes were in the ARG database, but some of them were not directly involved in the autophagy process. For example, GAPDH is a housekeeping gene involved in glycolysis, and Fos is a translation factor. However, GAPDH was recently found to be a pivotal and central regulator of autophagy under glucose deficient conditions, undergoing AMPK-dependent phosphorylation and nuclear translocation to activate Sirt1 deacetylase activity. Fos can regulate downstream apoptosis-related genes when phosphorylated *via* the JNK pathway during the autophagic response induced by endoplasmic reticulum stress. Because the number of differential genes is small, we included all ARGs expressed by TNBC in the prognosis analysis. Cox proportional hazard regression analysis revealed 10 key prognostic ARGs related to the overall survival of TNBC patients. In further multivariate survival analysis, eight of the ten genes were selected and used to construct a risk model capable of independent prediction of tumor prognosis. The reliability of the signature model was validated using a dataset downloaded from the GEO database. The genes *ITGA3*, *HSPA8*, *CTSD*, *ATG12*, *CLN3*, *ATG7*, and *MAP1LC3C* negatively influenced patient survival, whereas *WIPI1* may protect the patients from cancer-related death. Of note, tracing the clinical features of the tumors revealed that high *WIPI1* and *ATG12* expression levels were closely related to the stage of TNBC. However, in the differential expression analysis, no significant differences in the two genes were found between the TNBC and normal samples.

The autophagy response consists of four steps: (1) cytoplasmic components are captured by phagophores; (2) the phagophores expand and close to become autophagosomes; (3) the autophagosomes combine with vesicles or endosomes; (4) the autophagosomes fuse with lysosomes, the cargos are eliminated, and the products are recycled. Several ARGs regulate this process (Levine & Kroemer, 2019). In the first step of autophagy, serine/threonine-protein kinase (ULK) induces phagophore nucleation by phosphorylating the Beclin-1 complex. The subsequent phagophore elongation comprises two ubiquitin-like conjugation processes: the formation of the *ATG12–ATG5-ATG16L1* complex and the conjugation of light chain 3 (LC3) to phosphatidylethanolamine (Radoshevich et al., 2010). *ATG7* is one of the modulators of the above process (Tanida et al., 2012). PI3P production represents the initiation signal for autophagosome formation. The WIPI1 protein family specifically binds with PI3P at the nascent autophagosome to activate the PI3P signal, which controls early autophagosome assembly and is implicated in both canonical and noncanonical autophagy pathways (Proikas-Cezanne et al., 2015; Tsuyuki et al., 2014). The expression of *WIPI1* is strongly elevated in human cancers such as melanoma and colon cancer (D’Arcangelo et al., 2018), but the role of WIPI1 in the oncogenetic process of TNBC is unclear.

The degradation of selective substances is mediated by specific autophagic receptors that contain an *MAP1LC3/LC3*-interacting region motif. High levels of *MAP1LC3A* have been observed in many tumor cell lines and are associated with impaired autophagic activity, which facilitates carcinogenesis (Bai et al., 2012; Giatromanolaki et al., 2014). For breast cancer, cells expressing LC3A protein showed three distinct autophagic patterns and an increased number of “stone-like” structures were linked with a less favorable prognosis (Sivridis et al., 2010). In a study on drug sensitivity, *LC3A* was reported to be a biomarker for Lapatinib-resistance (Zhang et al., 2017). In our study, the expression level of LC3A in TNBC was lower than that in normal tissues, but the level reflected aggressive tumor behavior.

The ATG protein cathepsin D (encoded by *CTSD*) is a lysosomal, aspartic endoproteinase. Extracellular cathepsin D can modify the local extracellular matrix, inducing oncogenic activity by proteolysis at low pH or *via* protein-protein interactions. In a study involving 504 TNBC samples, *CTSD* was found to be overexpressed in 71.5% of the samples and was proposed to be a prognostic indicator for the TNBC outcome (Foekens et al., 1999). In the MDA-MB-231 metastatic xenograft model, the anti-cathepsin D antibody prevents M2-like macrophages and the recruitment of myeloid-derived suppressor cells, leading to less immunosuppression in the tumor microenvironment (Ashraf et al., 2019).

Integrin signaling is one of the autophagy mechanisms that promote tumor invasion (Hellyeh & Johanna, 2018). *ITGA3*, a receptor for fibronectin, laminin, and collagen, participates in focal adhesion, invadopodia formation, and matrix degradation. *ITGA3* has been reported to account for the differentiation and metastasis of many cancer types through extracellular matrix interactions, focal adhesion, and other molecular mechanisms. Targeting on *ITGA3* prevents cancer progression.

HSPA8 is involved in protein import into organelles or cellular compartments. When cells are exposed to stress, HSPA8 is notably induced and serves as a buffering system to maintain cell survival (Liu, Daniels & Cao, 2012; Stricher et al., 2013). In the cancer environment, it may activate immunocytes to promote tumor cell killing, but can also enhance immune system escape, enabling tumor cells to survive. HSPA8 overexpression has been found in a wide range of human cancers and is considered to be closely related to tumor invasion and metastasis (Xiang, You & Li, 2018).

*CLN3* impacts the function of lysosomes and galactosylceramide lipid transport from the Golgi apparatus to lipid rafts in the plasma membrane. Defects in the gene or low levels of *CLN3p* lead to enhanced neurodegeneration and apoptosis, while high levels of *CLN3p* may lead to the inhibition of apoptosis, thereby promoting carcinogenesis (Mirza et al., 2019). The expression of *CLN3* mRNA and CLN3 protein is increased in a variety of cancers, including breast cancer. In a study of human breast cancer samples, the absence of HER2 expression was found to be correlated with *CLN3* overexpression (Makoukji et al., 2015).

## Conclusions

In summary, TNBC is a very aggressive cancer type with unique genetic and molecular properties. Understanding the bilateral roles that autophagy genes play in TNBC development is fundamental for precise control of the disease. The present study, based on bioanalyses of data from the TCGA database, represents the first attempt to construct an ARG model to evaluate the survival risk for TNBC. The eight genes included in the model may serve as potential biomarkers or targets to optimize the treatment of TNBC. However, this study had limitations because we only analyzed the survival times of TNBC patients impacted by ARGs. We could not perform stratified analysis of the patients according to therapeutic strategies including chemotherapy and radiotherapy, which may have profoundly influenced the survival outcomes. Experiments on clinical samples are needed to validate our conclusions in the future.

##  Supplemental Information

10.7717/peerj.12878/supp-1 Supplemental Information 1 Raw data

## Additional Information and Declarations

### DNA Deposition

The following information was supplied regarding the deposition of DNA sequences: The TNBC microarray data (GSE38959) are available at GEO (Platform GPL4133).

### Data Availability

The following information was supplied regarding data availability: The raw data is available in the Supplementary File.
